# Sterilization of Water

**Published:** 1890-08

**Authors:** 


CHARLES G. CURRIER, M. D., NEW YORK, ON STERILIZATION OF WATER: *Conclusions*—Unless extraordinarily resistant, water bcomes sterilized if it be at or near the boiling temperature for fifteen minutes. If the same degree of heat be maintained for five minutes, all harmful micro-organisms will have been destroyed. Still less time serves to destroy the disease-producing varieties which are recognized as liable to occur in water. Thus merely raising to the boiling-point a clear water containing the micro-organisms of malarial disorders, typhoid, cholera, diphtheria, or of suppurative processes, and allowing it to gradually cool, insures the destruction of these germs. They are also destroyed by keeping the water for from a quarter of an hour to half an hour at a temperature of 70° C.

Occasionally, however, very resistant but harmless bacteria may get into water. The brief heating renders them safe for drinking purposes; but when it is desired to destroy every micro-organism that may be present in a contaminated water, it should be heated for one hour and allowed to cool slowly. Then it may be used for cleansing wounds or for alkaloidal solutions which will keep indefinitely, if no germs be introduced after the solution has been heated.—*Medical Record, June, 1890.*

